# Heterogeneity of Stomatal Pore Area Is Suppressed by Ambient Aerosol in the Homobaric Species, *Vicia faba*

**DOI:** 10.3389/fpls.2020.00897

**Published:** 2020-06-25

**Authors:** David A. Grantz, Marcus Karr, Juergen Burkhardt

**Affiliations:** ^1^Department of Botany and Plant Sciences, Kearney Agricultural Center, University of California, Riverside, Riverside, CA, United States; ^2^Institute of Crop Science and Resource Conservation, University of Bonn, Bonn, Germany

**Keywords:** aerosol, climate change, gas exchange, humidity, particulate matter, patchy stomata, vapor pressure deficit

## Abstract

Stomatal pore area is heterogeneous across leaf surfaces. This has been considered as “patchy stomatal conductance,” and may have substantial implications for photosynthetic efficiency. Aerosols have always been important elements of plant environments, but their effects on stomatal control of plant water relations, and stomatal heterogeneity specifically, have not been considered. Here we evaluate the spatial coordination of pore area in the glabrous and homobaric leaves of *Vicia faba* grown under two aerosol treatments and measured at four levels of VPD. We construct a large dataset (*n* > 88,000 discrete comparisons) of paired pore areas and distances between the pores. Plants were grown in ambient urban air and in filtered air (FA) to determine the effect of ambient aerosol on stomatal properties. Pore area exhibited spatial organization, as well as considerable variability among closely co-located pores. The difference between pore areas was positively correlated with the distance between the pores, in both aerosol treatments and at all VPDs. However, aerosol deposition reduced both the magnitude of variability between pores and the rate at which this variability increased with pore separation distance. These data support previous conclusions that deposition of hygroscopic aerosol may create a thin aqueous film across the leaf surface that connects neighboring stomata to each other and to the leaf interior. Aerosol impacts on stomatal heterogeneity and gas exchange are not adequately considered in current assessments of stomatal control.

## Introduction

Heterogeneous stomatal opening across the surface of individual leaves may result in stomatal “patchiness,” random variation, or spatially coherent trends in pore area across the surface. Stomatal heterogeneity has been observed in many species (Downton et al., 1988, 1990; Terashima et al., 1988; Sharkey and Seemann, 1989; Terashima, 1992; Pospisilova and Santrucek, 1994; Haefner et al., 1997; Weyers and Lawson, 1997; Beyschlag and Eckstein, 1998; Mott and Buckley, 1998, 2000). Non-uniform pore areas and resulting uneven conductance for CO_2_ and water vapor complicate standard measurements and calculations of gas exchange parameters. Heterogeneity observed at all stages of leaf elongation in *Rosa* shows that this is not a transient developmental feature (Fanourakis et al., 2015). Under conditions of high boundary layer conductance, stomatal heterogeneity may be detrimental to gas exchange efficiency, but under conditions of low wind or large leaves, which reduce boundary layer conductance, and under conditions of low overall stomatal conductance, heterogeneity may improve photosynthetic efficiency (Buckley et al., 1999). This reflects the effects of evaporative cooling of the leaf on fluxes and gradients of both water and CO_2_, and the transport efficiency of those pores that remain widely open. The significance and ubiquity of stomatal heterogeneity and its relationship with environmental conditions (Cheeseman, 1991; Gunasekera and Berkowitz, 1992; Cardon et al., 1994) as well as the role of aerosol deposition (Burkhardt and Grantz, 2017) in generating such heterogeneity have not been adequately considered.

Areas of coordinated stomatal behavior may range from a few mm to a few cm in extent (Terashima, 1992; Mott and Buckley, 1998, 2000; Mott and Peak, 2007) and are often bounded by veins, particularly in heterobaric leaves (Siebke and Weis, 1995; Haefner et al., 1997; Weyers and Lawson, 1997; Mott and Buckley, 2000). Patterns of heterogeneity can be transient, with patches of coherent stomatal response migrating within and even between areoles of heterobaric leaves (Kamakura et al., 2012), even though gas diffusion is restricted to areoles defined by vasculature with bundle sheath extensions (Terashima, 1992; Mott and Buckley, 2000).

Spatially coherent stomatal behavior is also observed in homobaric leaves (Kappen et al., 1987; Loreto and Sharkey, 1990; Mott and Parkhurst, 1991; Terashima, 1992; Eckstein et al., 1996; Mott and Buckley, 2000; Kamakura and Furukawa, 2008). This heterogeneity is less patchy and more characterized by trends across the leaf surface, from leaf base to tip (Nardini et al., 2008), margin to midrib (Weyers and Lawson, 1997), and more generally across the lamina (Terashima et al., 1988). Patches observed as ^14^CO_2_ fixation in homobaric *V. faba* (Terashima et al., 1988) were larger than in many heterobaric species and could be considered gradients across the leaf, with greater heterogeneity within the patches than in heterobaric species (Spence, 1987; Terashima et al., 1988). Stomatal aperture in homobaric *Commelina communis* (Smith et al., 1989) exhibited gradients as high as 3 μm per mm and 20 μm from leaf edge to midrib. This behavior may be observed even in epidermal peels exposed to uniform physical and chemical conditions. The distribution of pore opening often approximates a normal distribution (e.g., *V. faba*; Laisk et al., 1980). This variance within and between leaves obscures spatial patterns across individual leaves (Weyers and Lawson, 1997), so that heterogeneity in homobaric leaves remains poorly characterized.

Uneven levels of conductance across the surfaces of individual leaves have been associated with discrepancies in estimation of intercellular CO_2_ (C_i_). Values calculated from measured fluxes of CO_2_ and water vapor exceeded directly measured values by up to 15% (Buckley et al., 1999; Boyer, 2015a,b; Hanson et al., 2016). This suggested a pathway for water efflux that was unavailable to CO_2_ influx, potentially peristomatal transpiration from around the stomatal pores (Maier-Maercker, 1983; Grantz, 1990), or cuticular transpiration from across the epidermis between the pores (Kerstiens, 1996; Boyer et al., 1997; Hanson et al., 2016). Alternatively, stomatal heterogeneity could distort the calculation of C_i_ (Terashima et al., 1988) as measurements of steady state gas exchange reflect the leaf-wise average of highly dynamic heterogeneous individual pores and patches of pores (Siebke and Weis, 1995).

Increased evaporative demand (leaf to air vapor pressure difference; VPD) reduces stomatal conductance and increases stomatal heterogeneity (Sharkey and Seemann, 1989; Downton et al., 1990; Haefner et al., 1997). Experimentally imposed local changes in humidity at the leaf surface altered the apertures of stomata within the affected area, and those of stomata up to 0.4 cm away, outside of the directly affected area. The linkage was apparently through epidermal turgor, although this mechanism may operate most effectively over short distances (Haefner et al., 1997; Mott et al., 1997, 1999; Mott and Franks, 2001). Variability in mesophyll biochemistry (Osmond et al., 1999) and xylem water relations may become more effective at larger scales of leaf or branch (Buckley and Mott, 2000).

Aerosol deposition (Burkhardt and Grantz, 2017) may affect both the asymmetric flux pathways for water and CO_2_ (Grantz et al., 2018) and stomatal heterogeneity. Deposition of hygroscopic, particularly chaotropic, aerosol (Tsigaridis et al., 2006; Pringle et al., 2010; Burkhardt and Grantz, 2017) reduces surface tension and results in development of thin liquid films on leaf surfaces (Eiden et al., 1994; Dutcher et al., 2010; Burkhardt and Hunsche, 2013; Burkhardt and Grantz, 2017; Fernandez et al., 2017). The liquid films penetrate into stomatal pores (Eichert et al., 1998, 2008; Basi et al., 2014; Kaiser, 2014), providing a liquid phase linkage between the saturated leaf apoplast and the dry atmosphere. This pathway is not under diffusional (i.e., stomatal) control and thus water loss from this pathway through evaporation at the leaf surface increases as VPD increases, even in species such as *V. faba* that exhibit strong closing response to increasing VPD (Pariyar et al., 2013; Grantz et al., 2018). The presence of liquid films can be visualized by electrical conductance measurements (Burkhardt and Eiden, 1994; Burkhardt et al., 1999; Burkhardt and Hunsche, 2013) and by electron micrography (Burkhardt and Grantz, 2017; Burkhardt et al., 2018; Grantz et al., 2018). Aerosol deposition reduces stomatal apertures (Burkhardt et al., 2001a; Burkhardt, 2010; Burkhardt and Grantz, 2017; Grantz et al., 2018) while increasing both water flux (Burkhardt et al., 2001a; Grantz et al., 2018) and minimum (cuticular) leaf conductance (Burkhardt and Pariyar, 2014, 2016; Grantz et al., 2018).

We hypothesized that aerosol-induced surface moisture may link individual pores across the leaf surface by providing a more homogeneous hydraulic and vapor pressure environment, thereby reducing stomatal heterogeneity despite potential desiccating effects of liquid phase water loss and the previously documented reduction in pore area (Grantz et al., 2018). Here we characterize the distribution of stomatal pore areas, a subject of previous consideration (Laisk et al., 1980; Spence, 1987; Gorton et al., 1989; Terashima, 1992; Weyers and Lawson, 1997) and the role of aerosol deposition on this heterogeneity, which has not previously been considered. We analyze a previously available dataset of 3600 direct microscopic pore area measurements (Grantz et al., 2018) to create a novel database of 88,200 discrete pore to pore comparisons of distances between the pores (d) and the differences between their pore areas (ΔA). We evaluate the distribution of pore areas, the local and larger scale heterogeneity of stomatal opening, and the effect of four levels of VPD and two levels of ambient aerosol on these characteristics.

## Materials and Methods

### Plant Material

Plants of *Vicia faba* (L.) were grown from seed as described previously (Grantz et al., 2018) in plastic pots in greenhouses at the University of Bonn, Germany. Plants received complete nutrient solution (Ferty 3, Planta Düngemittel GmbH, Hohenstauf, Germany) weekly and irrigation as needed.

Plants were randomly assigned either to a greenhouse ventilated with ambient air (AA) or an adjacent greenhouse ventilated with filtered air (FA). Filtration removed nearly all particles (Pariyar et al., 2013; Grantz et al., 2018). The aerosol was typical of ambient particulate matter in central Europe (about 35% ionic; see Grantz et al., 2018 and references therein). Aerosol concentration in the AA greenhouse (cloud chamber condensation nuclei counter; TSI 3783; TSI, Shoreview, MN, United States) was 6–7 × 10^9^ particles m^–3^. This was reduced in the FA greenhouse by 99% to 5–10 × 10^6^ m^–3^; confirming previous measurements (e.g., Pariyar et al., 2013). Other environmental parameters including temperature, relative humidity and concentrations of ozone were similar in the greenhouses (see Supplementary Figures S1, S2 in Grantz et al., 2018). Plants were exposed to natural daylength and sunlight (approximately 70% of ambient irradiance).

Measurements were obtained 5 weeks after planting on one leaflet of leaf 5 or 6 (youngest fully expanded; mean leaflet area about 32 cm^2^) when plants had been exposed for 3–4 weeks in the greenhouses. Leaflets remained attached to intact plants during all measurements.

### Measurement of Pore Area at Controlled VPD

Plants were transported from Bonn to Kiel, Germany. Pore area measurements (see Grantz et al., 2018 for further details) were obtained while leaf to air VPD was controlled in a flow-through gas exchange system with an integrated inverted video microscope (Kaiser and Kappen, 1997, 2000; Kaiser, 2009). The gas exchange cuvette was held at constant temperature (25°C ± 0.1°C) and irradiance (PPFD = 450 ± 25 μmol m^–2^ s^–1^). Dew point was held at 23.15, 19.0, 14.0, and 5.0°C (±0.05°C), yielding VPD of 0.33, 0.97, 1.6, and 2.3 kPa and RH of 90%, 68%, 50%, and 27%, at abaxial leaf temperature (25°C; Type K thermocouple, 0.075 mm). Air circulation in the cuvette (1 m s^–1^) yielded laminar boundary layer conductance of 1300 mmol m^–2^ s^–1^ (Kaiser, 2009).

Nine plants were used for each of the AA and FA treatments (Experiment 1, Grantz et al., 2018). Measurements on plants subjected to the two aerosol treatments were alternated on successive days. Pore area was measured on 50 stomata per leaflet, on the abaxial surface, within the 1 cm^2^ area of a predefined grid. The sample area was located away from major veins and leaf margins. The 50 stomata represented about 0.2% of the stomata in the 1 cm^2^ viewing area. The location (pore center) and focus depth (narrowest part of the pore center) of each pore was stored electronically (Kaiser, 2009) to allow rapid re-imaging at each VPD.

### Data Analysis

Data were stored with plant, leaf and pore identifiers, pore area, and pore coordinates in the Cartesian framework defined across the microscope stage (Kaiser, 2009). The area of each pore was determined as an idealized ellipse determined by the directly measured length and width of each pore, taken as the major and minor axes (Grantz et al., 2018). The difference in pore area (ΔA) between pairs of stomata was evaluated as a function of their separation distance (d) across the leaf surface.

Values of d were calculated from the coordinates of the center of each pore using the Pythagorean Theorem executed in a custom Python program (v. 3.6; Python Software Foundation^1^). Unique pair comparisons [C(50,2) = (50)(50-1)/2 = 1225 per leaflet] were evaluated. With 9 leaflets/aerosol treatment, 2 treatments, and 50 pores/leaflet, there were 900 unique pores and with 4 levels of VPD there were 88,200 unique comparisons of ΔA vs. d. The maximum value of d was about 1.4 cm, representing the diagonal of the 1 cm^2^ square examined on each leaf.

Regressions of ΔA on d were calculated in Sigma Plot, v. 13.0 (Systat Software, San Jose, CA, United States; Regression Wizard). Normality [Kolmogorov–Smirnov (K–S) Test] and equality of variance (Brown–Forsythe Test) were generally not satisfied (*P* < 0.050). The sole exception was FA at low VPD, which was normally distributed. Mean ΔA (Table 1) and regression coefficients (Table 2) were evaluated by Two-Way ANOVA (Sigma Plot) with mean separation using the Holm-Sidak Multiple Range Test.

**TABLE 1:** Means of the differences between pore areas (ΔA).

	Mean difference in pore area (ΔA; μm^2^)		
VPD	Ambient Air (AA)	Filtered Air (FA)	AA vs. FA
0.33 kPa	37.6 ± 0.32 **(a)**	47.6 ± 0.37 **(b)**	*P* < 0.001
0.97 kPa	42.5 ± 0.36 **(c)**	56.1 ± 0.45 **(d)**	*P* < 0.001
1.6 kPa	43.6 ± 0.37 **(d)**	53.6 ± 0.43 **(c)**	*P* < 0.001
2.3 kPa	39.9 ± 0.39 **(b)**	45.7 ± 0.40 **(a)**	*P* < 0.001

*Lower case letters within columns refer to differences between VPD levels within each aerosol treatment. *P* values in the right hand column refer to differences between aerosol treatments within VPD levels. Means were analyzed by Two-Way ANOVA with mean separation (right hand column) by the Holm-Sidak method. The ΔA × VPD interaction was generally significant.*

**TABLE 2:** Linear regression coefficients of the relationships between the difference in pore area (ΔA) and the distance between the pores (d).

VPD	Ambient Air (AA)	Filtered Air (FA)	AA vs. FA
**(A) Intercept (μm^2^)**			
0.33 kPa	35.2 ± 0.75 **(a)** [*P* < 0.0001]	40.4 ± 0.88 **(a)** [*P* < 0.0001]	*P* < 0.001
0.97 kPa	36.8 ± 0.85 **(a)** [*P* < 0.0001]	48.7 ± 1.07 **(b)** [*P* < 0.0001]	*P* < 0.001
1.6 kPa	41.0 ± 0.87 **(b)** [*P* < 0.0001]	48.2 ± 1.01 **(b)** [*P* < 0.0001]	*P* < 0.001
2.3 kPa	36.6 ± 0.93 **(a)** [*P* < 0.0001]	44.4 ± 0.94 **(c)** [*P* < 0.0001]	*P* < 0.001
**(B) Slope (μm^2^ cm^–1^)**			
0.33 kPa	4.6 ± 1.34 **(a)** Adj *r*^2^ = 0.0010 [*P* = 0.0007]	13.6 ± 1.50 **(a)** Adj *r*^2^ = 0.0072 [*P* < 0.0001]	*P* < 0.001
0.97 kPa	11.1 ± 1.51 **(b)** Adj *r*^2^ = 0.0048 [*P* < 0.0001]	14.1 ± 1.84 **(a)** Adj *r*^2^ = 0.0052 [*P* < 0.0001]	*P* = 0.208
1.6 kPa	5.1 ± 1.55 **(a)** Adj *r*^2^ = 0.0009 [*P* = 0.001]	10.3 ± 1.74 **(a)** Adj *r*^2^ = 0.0031 [*P* < 0.0001]	*P* = 0.026
2.3 kPa	6.4 ± 1.66 **(ab)** Adj *r*^2^ = 0.0013 [*P* = 0.0001]	2.5 ± 1.62 **(b)** Adj *r*^2^ = 0.0001 [*P* = 0.1188]	*P* = 0.093

**P* values in brackets refer to the individual regression coefficients. Lower case letters within columns refer to differences between VPD levels within each aerosol treatment. *P* values in the right hand column refer to differences between coefficients for each aerosol treatment within VPD levels. Coefficients were analyzed by Two-Way ANOVA with mean separation by the Holm-Sidak method.*

## Results

### Pore Location

**FIGURE 1:**
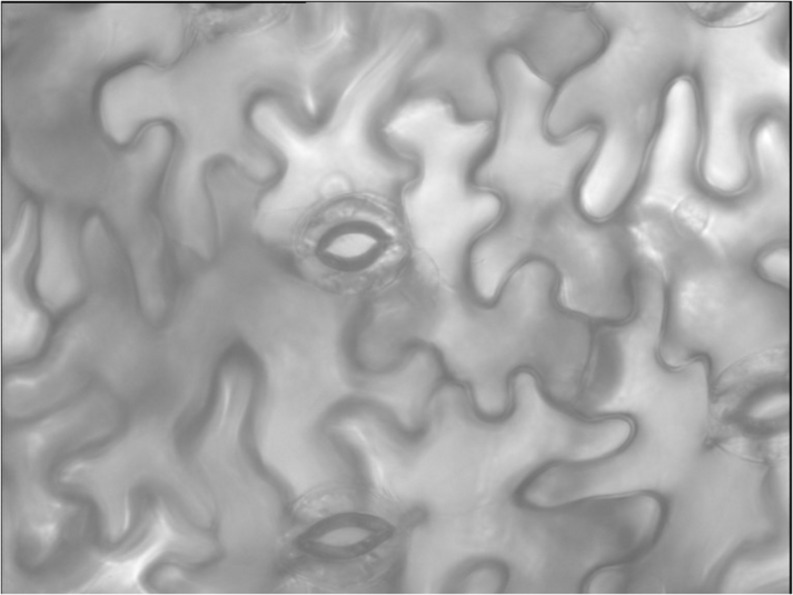
Typical stomatal pores whose areas (A) were measured. Representative example at low VPD (0.33 kPa). Micrograph dimensions 300 × 300 μm.

The imaging system facilitated collection of a large number of clear photomicrographs (Figure 1). At all levels of stomatal opening, both within each VPD and across the levels of VPD, the pores were well characterized as ellipses, allowing the area (A) of each pore to be calculated from the directly measured length and width. Leaves of *V. faba* exhibited uniformly kidney-shaped guard cells (Figure 1), lacking specialized stomatal subsidiary cells and the bundle sheath extensions that play a role in stomatal patchiness in heterobaric species.

**FIGURE 2:**
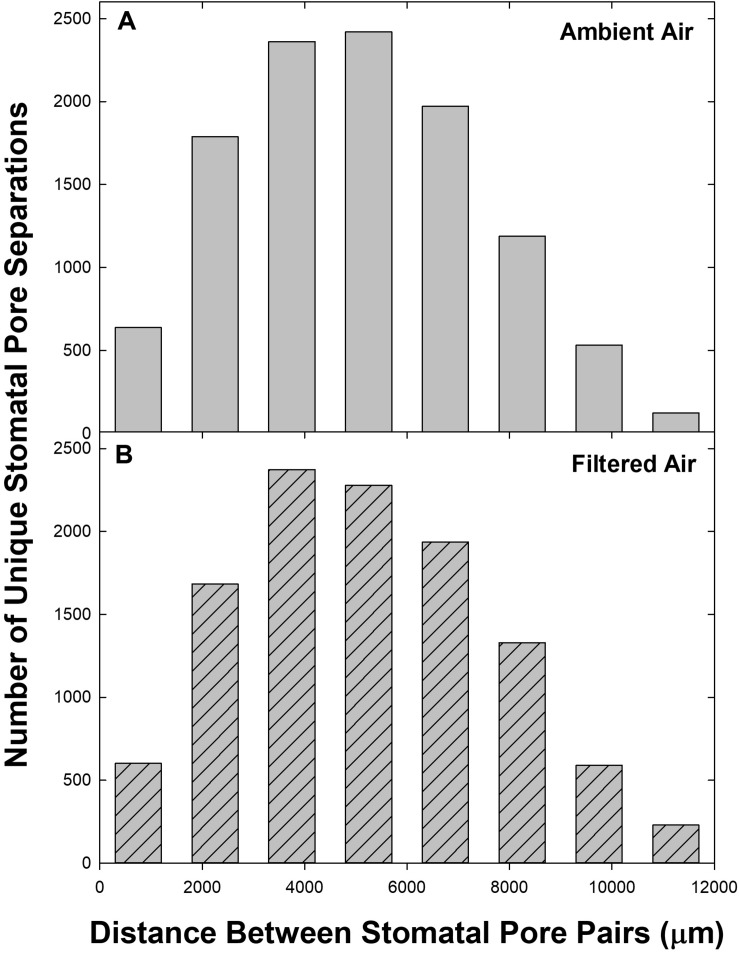
Frequency distribution of pore separations (d) among the 50 stomata measured on each of 9 leaves (*n* = 11,025 per panel). Distance classes: 0 ≤ *d* < 1500 to 10500 ≤ *d* < 12000, centered at 750–11250. For *d* ≥ 12000 (*n* = 20 for AA and 55 for FA) frequencies were pooled with 10500 ≤ *d* < 12000. **(A)** Ambient air; **(B)** filtered air.

The distances (d) between pores were distributed approximately normally (Figure 2). Neither the shape of the distribution nor the magnitude of the distance scale differed between leaves exposed during leaf development to ambient aerosol (AA) or to FA (cf. Figures 2A,B). These characteristics were also stable across the levels of VPD (not shown), despite the potential for leaf shrinkage due to reduced leaf water content (not measured) at elevated VPD.

### Pore Area

**FIGURE 3:**
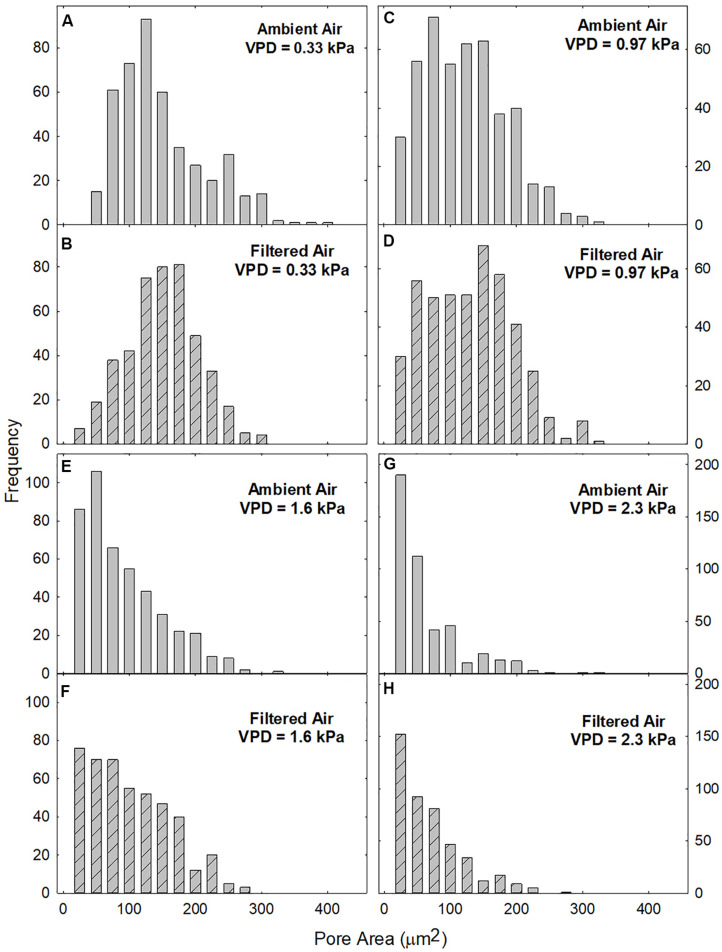
Frequency distribution of pore areas (A) at different levels of evaporative demand (VPD), in leaves exposed to ambient (gray bars; **A,C,E,G**) or filtered (hatched bars; **B,D,F,H**) air. Area classes: 0 ≤ A < 50 to 400 ≤ A < 450, centered at 25–425. The smallest visible bars represent 1 pore.

The large data set available for this study allowed a robust characterization of the distributions of pore area (A) in the two aerosol treatments over a range of VPDs (Figure 3). At low VPD (0.33 kPa) the distributions of A in both AA (Figure 3A) and FA (Figure 3B) treatments were approximately bell shaped, although only the FA treatment (Figure 3B) formally satisfied the Kolmogorov–Smirnov test for normality (K–S statistic of 0.661). All other distributions exhibited K–S < 0.001.

The distributions of A were similar in AA and FA leaves at all levels of VPD (cf. Figures 3A–H). VPD did not differ during plant growth between the AA and FA treatments but was varied experimentally during measurement of pore areas. Skewing toward the origin increased with VPD (Figure 3) so that the quasi-bell shaped distribution was no longer evident at 1.6 kPa (Figures 3E,F) or 2.3 kPa (Figures 3G,H) in either aerosol treatment.

**FIGURE 4:**
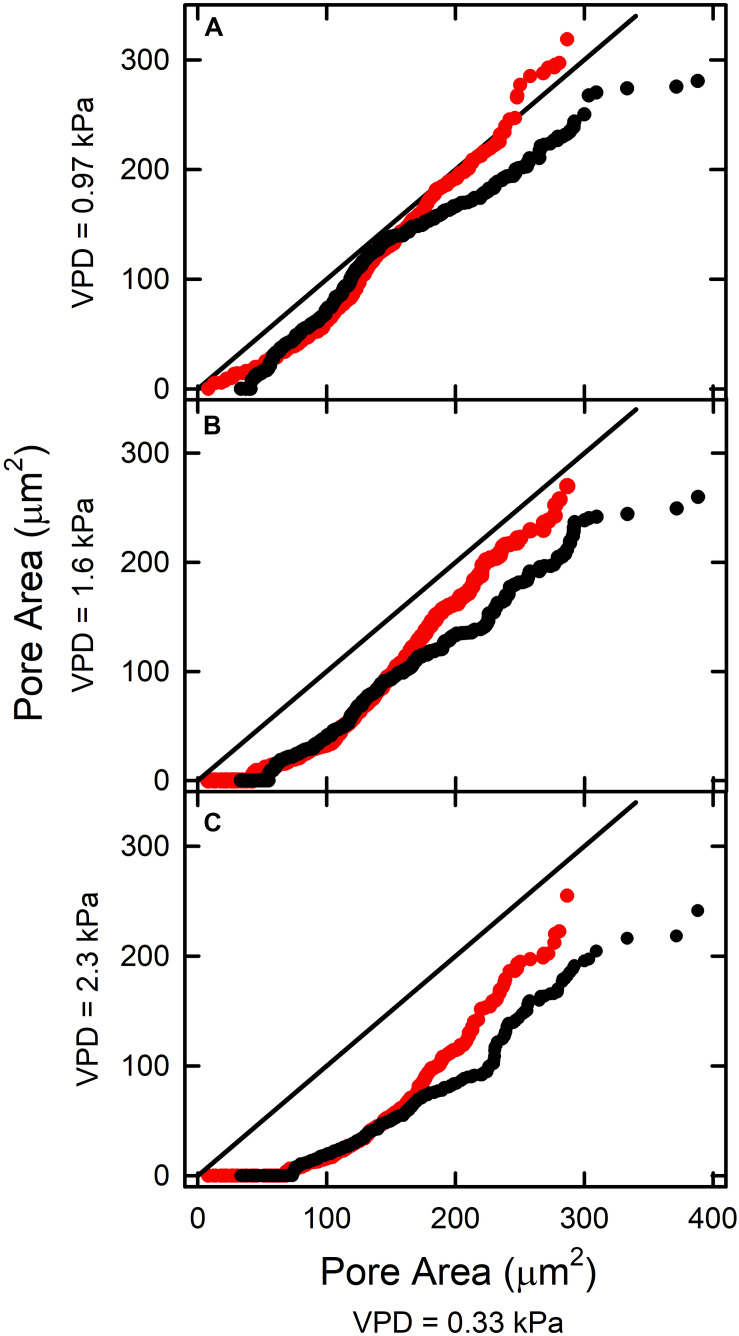
The relationship between stomatal pore area at elevated VPD [**(A)** 0.97 kPa; **(B)** 1.6 kPa; **(C)** 2.3 kPa] and pore area measured at low VPD (0.33 kPa). The AA treatment is shown in black, and the FA treatment in red. The solid line represents the 1:1 line.

In most cases, increasing VPD reduced pore area, i.e., with greater deviation below the 1:1 line (Figure 4). In FA at 0.97 kPa the pattern was different. The less open pores closed in response to increased VPD, but pores initially exhibiting A > 190 μm^2^ (Figure 4A; red circles) did not close below the initial A. Individual stomata behaved consistently over the range of VPDs. Those with small pore areas at 0.33 kPa exhibited small areas at higher VPD. Many that were only slightly open at low VPD closed completely at higher VPD. With increasing VPD, the number of such stomata and their initial pore areas increased (horizontal part in lower left end of each curve; Figure 4).

Leaves exposed to ambient aerosol also exhibited greater skewing toward the origin. This reduction in magnitude was reflected in reduced mean ΔA between pores in AA relative to FA observed at all levels of VPD (Table 1). In general, the AA treatment closed more substantially than the FA as VPD was increased, particularly at greater initial pore area at low VPD (Figure 4). Among stomata with areas below 180 μm^2^ at 0.33 kPa, there was little effect of aerosol on the relationships between pore area at low VPD and at higher VPD. This point of divergence between AA and FA was similar at all VPD (Figures 4A–C). Above this value of initial opening, pore areas in FA and AA diverged, with AA smaller than FA. This was observed from 0.33 kPa to 0.97 kPa (Figure 4A), even though the response of FA to VPD was minimal among the more open pores. The sensitivity to aerosol increased with pore area observed at low VPD, but did not change substantially with increasing VPD.

### Stomatal Heterogeneity

**FIGURE 5:**
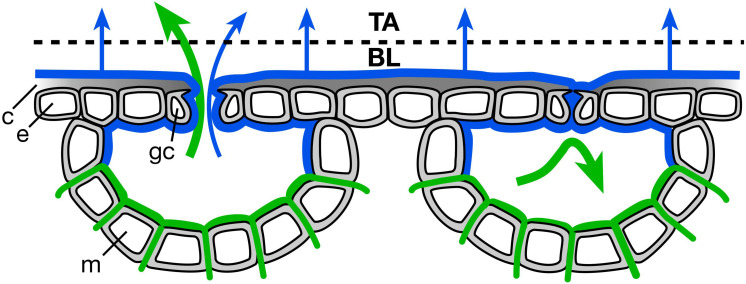
The hypothesized role of thin liquid films in reducing microenvironmental variability across the leaf surface. Blue lines represent liquid formed by condensation to deposited hygroscopic aerosol on the leaf surface, including that which penetrates the stomatal pores; blue arrows represent the evaporative flux of water derived from this liquid. Green lines represent conventional apoplastic liquid water derived from the soil-root-xylem pathway passing through and around mesophyll cells (m); green arrows represent the much larger evaporative flux of this water when stomata are open (left side) and its elimination by stomatal closure (right side). Evaporation occurs from aerosol-condensed water with both open (left side) and closed (right side) stomata, as liquid continuity between leaf interior and exterior is maintained largely independently of stomatal pore area determined by guard cell (gc) turgor. The leaf boundary layer (BL) is uniformly humidified across the leaf surface and the osmotic environment of the cuticle (c) and underlying epidermis (e) is made more homogeneous by the presence of a liquid film. All fluxes (blue and green arrows) eventually pass through the BL and enter the mixed free atmosphere (TA), representing water loss from the leaf.

We had hypothesized that stomatal heterogeneity, both ΔA between closely co-located pores and the increase of ΔA with d between more distant pores, would be reduced by aerosol deposition. The suggested mechanism, involving liquid films on the leaf surface, is illustrated in Figure 5 showing an open stoma at the left and a closed stoma at the right. Regardless of pore area, water evaporates from the surface of the leaf (blue arrows), potentially drawing both from the surface water (Figure 5; blue lines) and from soil, tissue, and apoplastic storage (continuity indicated by green lines). Conventional transpiration from the apoplast (green arrows) occurs through the open pore (left side) but this diffusive transport is almost completely blocked by stomatal closure (right side).

In contrast, a continuous liquid film developing from deliquescence of deposited hygroscopic aerosol may spread across large areas of the leaf and penetrate into the stomatal pores. This liquid path through the pore reduces stomatal control of water loss. This may degrade epidermal water status by enhancing water loss, while also reducing the variability in the hydraulic and humidity environments across the leaf surface (Figure 5). While reduced epidermal water content might increase stomatal heterogeneity, the uniform environment under the liquid film might lead to more uniform epidermal water status and reduce heterogeneity.

**FIGURE 6:**
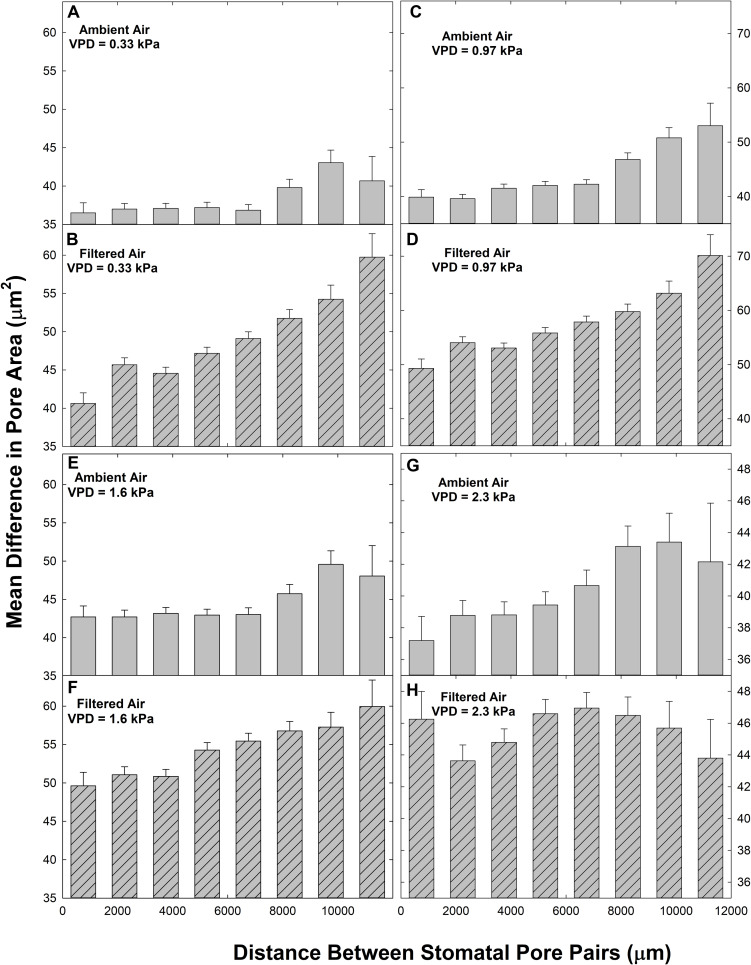
Relationships between the paired difference between pore area (ΔA) and the distance separating the pores (d) in ambient air (AA; gray bars; **A,C,E,G**) and filtered air (FA; hatched bars, **B,D,F,H**) at different levels of evaporative demand (VPD), presented as mean ± s.e. Distance classes as in Figure 2.

**FIGURE 7:**
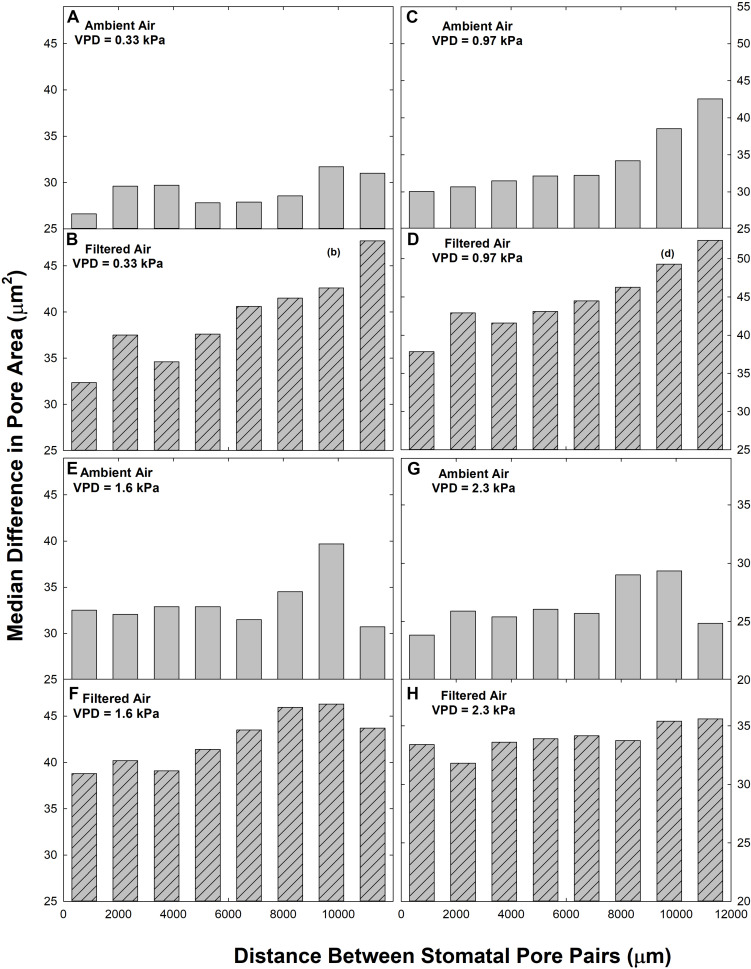
Relationships between the paired difference between pore area (ΔA) and the distance separating the pores (d) in ambient air (AA; gray bars; **A,C,E,G**) and filtered air (FA; hatched bars, **B,D,F,H**) at different levels of evaporative demand (VPD), presented as median. Distance classes as in Figure 2.

We observed a reduction in heterogeneity in the ambient aerosol treatment. The intercept of ΔA on d was significantly lower in AA than FA at all VPD (Table 2A), reflecting reduced heterogeneity among closely co-located pores (extrapolated to zero separation). Exposure to aerosol also significantly reduced both mean ΔA (Table 1 and Figure 6) and median ΔA (Figure 7) at all levels of VPD. The considerable local variability is illustrated by the closely positioned pores in Figure 1, exhibiting ΔA of about 25% despite the guard cells bordering on the same epidermal cell.

Although there were fewer pores at small and large separations (Figure 2), the distributions of both mean ΔA (Figure 6) and median ΔA (Figure 7) revealed a consistent increase with d over the entire range. This was observed in both AA and FA leaves, but the increase in ΔA with d was reduced by aerosol (Figures 6, 7). The slope of ΔA on d was significantly reduced in AA relative to FA at VPD of 0.33 kPa and 1.6 kPa (Table 2B; c.f. Figures 6G,H, 7G,H). Slopes did not differ at 0.97 kPa or 2.3 kPa. This reflects a general reduction in the increase in heterogeneity with distance within the 1 cm^2^ sample area caused by aerosol exposure. The median analysis is presented, along with the mean, because the data often varied from normality.

The impact of VPD was less consistent. In AA, mean ΔA (Table 1) and the intercept of ΔA at *d* = 0 (Table 2A) increased with initial increase of VPD from 0.33 kPa through 0.97 to 1.6 kPa but declined at 2.3 kPa. In contrast, in FA, the mean and intercept of ΔA increased with VPD from 0.33 to 0.97 kPa, but began to decline at 1.6 kPa and declined further at 2.3 kPa. The pattern of response of ΔA over all pores (mean) and among closely spaced pores (intercept) was similar in the aerosol treatments, but the decline began at lower VPD in the absence of aerosol. In contrast, the slope of ΔA on d, reflecting larger scale heterogeneity, did not differ consistently between levels of VPD (Table 2B).

Heterogeneity of stomatal pore area was composed of two components, variability among pore areas that was not related to the distance between the pores (Table 2A; left-most bars, Figures 6, 7), and variability which trended systematically with d (Table 2B; distributions, Figures 6, 7). The first type of variability was expressed both as local heterogeneity between closely co-located pores, and as a component of the magnitude of ΔA between more distant pores. Large values of ΔA (>35 μm^2^) were observed among neighboring pores in both AA and FA. In all cases, local variability in ΔA among neighboring pores was greater than the increase in ΔA with d across the observation area of 1.4 cm (Figures 6, 7).

## Discussion

This study had two objectives, to characterize the distribution of individual pore areas over a range of VPD levels, and to elucidate the role of ambient aerosol in stomatal regulation of plant water relations. The first objective is advanced through analysis of an unusually large number of non-destructively imaged pore areas, acquired previously (Grantz et al., 2018). Previous experiments have approached this objective by imaging many fewer pores, either the same pores repeated over time in epidermal peels (Gorton et al., 1989) or different pores at different times in leaf impressions (Smith et al., 1989). We advance the second objective with a novel analysis of aerosol impacts on stomatal heterogeneity. We showed previously that transpiration per unit stomatal aperture and minimum leaf conductance both increased following aerosol deposition (Burkhardt et al., 2001a; Grantz et al., 2018). No previous analyses have considered whether aerosol deposition might also affect the uniformity of stomatal opening.

### Pore Area Distribution

Stomatal opening is characterized by a strong element of randomness that often results in a quasi-normal (bell-shaped) distribution of pore areas (Gorton et al., 1989; Terashima, 1992; Weyers and Lawson, 1997). Our observations confirm earlier reports, including in *V. faba* (Laisk et al., 1980; Kappen et al., 1987; Spence, 1987), of large within-leaf variability with quasi-normally distributed pore areas, particularly at VPD = 0.33 kPa and 0.97 kPa. There was large heterogeneity even among nearly contiguous pores, as observed previously in *V. faba* and other species. For example, adjacent stomata of homobaric tobacco (*Nicotiana tabacum*) ranged from fully closed to fully open (Pospisilova and Santrucek, 1994).

At higher VPD (1.6 kPa and 2.3 kPa), the distributions skewed toward the origin, with a larger proportion of closed pores and reduced mean and median values, as described previously (Grantz, 1990; Grantz et al., 2018). These highly skewed distributions may also belong to theoretically normal distributions that extend into the imaginary territory of negative pore area (Laisk et al., 1980). The maintenance of the bell-shaped distribution even as mean and median pore area declined, reflects synchronous, parallel, and potentially coordinated responses, of similar magnitude by pores of different initial areas and in different locations across the leaf (Saxe, 1979; Spence et al., 1983; Kappen et al., 1987; Spence, 1987). This coherent behavior resembles an emergent property (Mott and Buckley, 2000; Mott and Peak, 2007) but the mechanism of such coordination remains unknown. Elevated VPD increases transpiration and degrades epidermal water status, suggesting metabolic and hydropassive responses of guard cells to epidermal water relations (Mott and Franks, 2001; Buckley, 2005, 2019).

The deposition of hygroscopic aerosol on the leaf surface may act similarly. The presence of thin liquid films lining stomatal pores provides a non-diffusive liquid pathway for water loss (Burkhardt et al., 2001b; Grantz et al., 2018), potentially increasing transpiration and degrading epidermal water status. Hygroscopic, particularly chaotropic, aerosol enhances the formation of such films by deliquescence and facilitates their spread across the leaf surface and into stomatal pores by reducing the surface tension of the liquid on the leaf surface (Monteith, 1957; Eiden et al., 1994; Dutcher et al., 2010; Burkhardt and Hunsche, 2013; Burkhardt and Grantz, 2017; Fernandez et al., 2017). Electrical conductance measurements (Burkhardt and Eiden, 1994; Burkhardt et al., 1999; Burkhardt and Hunsche, 2013) and electron micrography (Grantz et al., 2018) demonstrate these films and their penetration into stomatal pores (Eichert et al., 1998, 2008; Basi et al., 2014; Kaiser, 2014). The resulting hydraulic linkage of apoplast to the leaf boundary layer is associated with a reduction of mean pore area and skewing of areas toward the origin (Burkhardt et al., 2001a, 2012; Burkhardt, 2010; Pariyar et al., 2013; Grantz et al., 2018).

The current study demonstrates an apparent 3-way synergy between elevated VPD, initial stomatal opening at low VPD, and deposition of aerosol, consistent with a cumulative effect on epidermal water relations. At pore areas above about 180 μm^2^ (at VPD = 0.33 kPa) the dynamics of AA and FA differ as VPD increased. The role of initial opening may reside in the conditions required to sustain liquid water on the leaf surface. This depends on the humidity of the BL which is substantially affected by transpiration. The role of aerosol deposition is to induce water accumulation on the leaf surface from unsaturated air, which becomes more significant at elevated VPD. In the absence of aerosol (FA), condensation on the leaf surface requires approximately 100% RH, but the presence of hygroscopic aerosol (AA) reduces this requirement substantially (e.g., to 75% RH for deliquescence of NaCl aerosol; Burkhardt and Grantz, 2017).

### Pore Area Heterogeneity

*Vicia faba* is homobaric, without bundle sheath extensions or specialized stomatal subsidiary cells. This removes within-areole coordination of gaseous and water potential environments and suggests weaker coordination among stomata at local scale and potentially greater coordination at larger scale than in heterobaric species. In the present study, variability among closely co-located pores was large. This baseline level of ΔA was a dominant component of mean and median ΔA at all levels of pore separation and at all levels of VPD. At larger scale, there was a consistent increase of ΔA with d at all VPD and both aerosol treatments. However, the increase in ΔA with distance was less than the local variability observed between closely spaced stomata. This reflects poor coordination between individual pores that was degraded further with greater separation within the 1.4 cm scale of our observations.

Degraded epidermal water status is associated with increased stomatal heterogeneity (Sharkey and Seemann, 1989; Downton et al., 1990; Haefner et al., 1997). However, in the present study, VPD did not consistently increase the magnitude of ΔA among neighboring pores, increasing only at moderate levels then declining.

The increased water loss per unit stomatal opening observed in the AA treatment (Grantz et al., 2018), suggested that aerosol could increase stomatal heterogeneity. However, this was not observed. Aerosol decreased heterogeneity among neighboring pores at all levels of VPD and reduced the rate of increase of ΔA with d at all VPD.

The liquid film at the leaf surface in the presence of aerosol may link stomata over potentially large areas of the leaf surface by making the near-surface micro-environment more homogeneous. This unifying effect may be more significant than the effect of the liquid film on increased water loss and potential impacts on epidermal water status.

Theories of stomatal optimization (Cowan and Farquhar, 1977) and of cavitation avoidance (Sperry et al., 2017), implicitly consider stomata as a population, whether at the leaf, branch or larger scale (Mott and Peak, 2007). Under conditions of high boundary layer conductance, heterogeneity may be detrimental, but in low wind or with large leaves it may improve gas exchange efficiency. This may explain similar growth of plants grown in AA or FA conditions (unpublished observations) in the slowly ventilated greenhouse, but degraded water use efficiency of AA leaves in the rapidly stirred cuvette of a gas exchange system (Pariyar et al., 2013). By reducing heterogeneity, aerosol deposition could reduce the errors in calculated values of C_i_, potentially a factor that distinguishes gas exchange measurements made under controlled environment conditions from those made in more aerosol-rich field conditions.

## Conclusion

Stomatal heterogeneity and patchy conductance remain enigmatic (Gunasekera and Berkowitz, 1992). Stomatal patchiness represents stronger stomatal coordination at local scale and poorer coordination at larger scale than predicted by true randomness (Sharkey and Seemann, 1989; Downton et al., 1990; Haefner et al., 1997; Lawson et al., 1998; Mott and Buckley, 2000; Mott and Peak, 2007). While heterogeneity among individual pore areas has been considered as noise (e.g., Cheeseman, 1991) it has more recently been seen as informative regarding the physiological bases of stomatal opening mechanics (Laisk et al., 1980), responses to environment (Mott et al., 1997; Mott and Buckley, 2000) and of the signal processing required for attainment of quasi-optimal stomatal behavior (Cheeseman, 1991; Cardon et al., 1994; Siebke and Weis, 1995; Kaiser and Kappen, 1997; Mott and Peak, 2007). Environmental drivers of coordination among individual members of populations of stomatal pores, including light and VPD, have been considered critical components of the stomatal regulatory system. Aerosol deposition to leaves has not been considered as often, but may play an important role.

We demonstrate, using a very large data set, that stomatal pore areas may be described as quasi-normally distributed, even as mean and median values change with VPD and aerosol deposition. We show that VPD and aerosol deposition both reduce mean pore area and the differences among pores, both at local and greater scale within individual leaves of homobaric *V. faba*. Synergy of these two ubiquitous environmental factors with the previously unexplored factor of initial stomatal opening, suggests linkage through impacts on epidermal water relations. The aerosol exposure in this experiment was typical of regional European particulate pollution (Putaud et al., 2010), and the study material a short-lived herbaceous annual. These effects may be greater in more hirsute and longer-lived leaves, and in more heavily polluted environments (Kuki et al., 2008; Yang et al., 2015). Aerosol impacts on leaf water relations and gas exchange, mediated by the degree of stomatal opening and its heterogeneity, may be more significant than commonly realized. Further research focused on the control elements regulating stomatal pore area (e.g., Buckley, 2019) and on their interactions with aerosol deposition (e.g., Burkhardt and Grantz, 2017) may be increasingly relevant to characterization of local and global budgets of water and carbon.

## Data Availability Statement

The datasets generated for this study are available on request to the corresponding author.

## Author Contributions

DG and JB conceived of the experiments. DG conducted the gas exchange and pore dimension experiments and analyzed the data. MK developed the software for spatial analysis. All authors contributed to interpretation of the results and wrote the manuscript.

## Conflict of Interest

The authors declare that the research was conducted in the absence of any commercial or financial relationships that could be construed as a potential conflict of interest.

## Footnotes

1 https://www.python.org/
